# More Chemicals Show Epigenetic Effects across Generations

**DOI:** 10.1289/ehp.120-a228

**Published:** 2012-06-01

**Authors:** Bob Weinhold

**Affiliations:** Bob Weinhold, MA, has covered environmental health issues for numerous outlets since 1996. He is a member of the Society of Environmental Journalists.

If jet fuel and synthetic pesticides had existed during your great-grandmother Irene’s pregnancy, you might have inherited a potential for infertility that could make you the last in your branch of the family tree. But if the science of transgenerational epigenetic biomarkers continues to develop, researchers may at least be able to track the cause of your infertility to her exposure to those agents. Those are two highlights from the latest of a series of studies by Michael Skinner, a professor of reproduction and environmental epigenetics, and his Washington State University colleagues.^1^

Epigenetic changes occur when the function of a gene is altered by various mechanisms although its DNA sequence remains stable. Transgenerational effects result from a mother’s exposure and are inherited through successive generations in the absence of direct exposure of the offspring.^2^ Such environmentally induced effects have been demonstrated in people, rodents, birds, fish, insects, worms, plants, and microbes, in some cases lasting dozens of generations.^3^

In the current study Skinner’s team administered daily injections to female rats during days 8–14 of gestation, the period of embryonic gonadal determination. The investigators exposed them to representatives of four classes of chemicals, each of which has a different signal transduction system: a plastics mixture, a pesticide mixture, a dioxin (2,3,7,8-tetrachlorodibenzo-*p*-dioxin, TCDD), and a hydrocarbon product (JP-8 jet fuel).^4^

The selected chemicals represent a range of substances people are regularly exposed to in military and civilian settings. There is published evidence of transgenerational inheritance effects linked with the plastics additive bisphenol A and for TCDD, but not for the other chemicals tested, says Lisa Helbling Chadwick, a health scientist administrator with the National Institute of Environmental Health Sciences (NIEHS), whose purview includes transgenerational epigenetics. (The NIEHS cofunded the study with the U.S. Department of Defense.)

The doses used in the studies (there were two for the plastics mixture and one for each of the other exposures) were deliberately higher than typically found in the environment. “We hit [the rats] with a hammer so we could see what the end point was,” Skinner says, adding that more research is needed to determine the implications for real-world human doses, exposure pathways, timing, mixtures, and other factors. Another unknown is how potential endocrine disruption at low doses may relate to any epigenetic impacts.

The team evaluated several reproductive system effects and discovered numerous statistically significant outcomes in the third successive (F_^3^_ ) generation. All four chemical classes significantly decreased the number of ovarian primordial follicles by approximately 30–40%, an effect that could result in impaired reproduction. Compared with controls, the plastics mixture, JP-8, and TCDD were associated with onset of puberty about two days earlier in females (roughly comparable to two years earlier in humans, Skinner says), and with serum testosterone concentration reductions of some 50–65% in males. The same substances were also linked with about a 20% increase in the anogenital distance index in females.^5^ The lower dose of the plastics mixture was associated with a roughly 15% increase in the male anogenital distance index. JP-8 was linked to about a 20% increase in sperm cell apoptosis.

**Figure:**
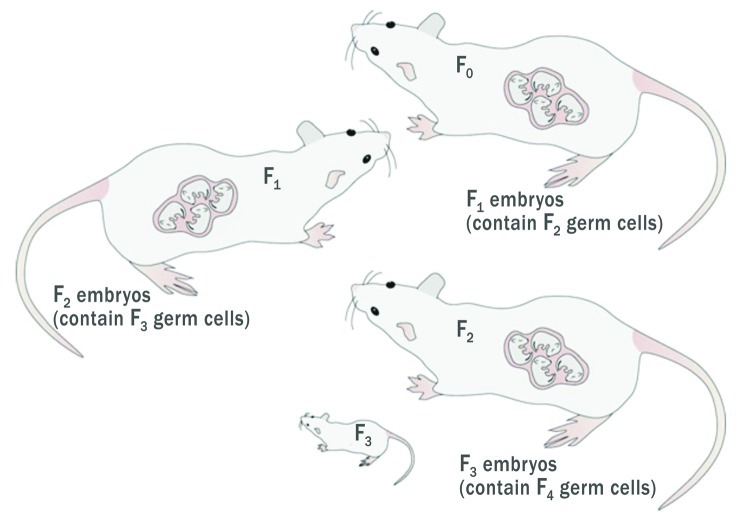
When a pregnant female (F0) is exposed to an agent, there is also direct exposure to her fetus (F1) and to the second successive generation (F2) that exists as developing germ cells within the F1 animal. F3 represents the first generation with no direct exposure. Walker and Gore (2011); doi:10.1038/nrendo.2010.215

The team also iden-tified in the sperm epi-genome unique DNA methylation regions for each chemical class, suggesting it may eventually be possible to retroactively track effects of specific chemicals.

The results are convincing for John McCarrey, a professor of cell and molecular biology at the University of Texas at San Antonio, who has been studying epigenetics for about 30 years and has an ongoing collaboration with Skinner, although not on this study. “[Skinner et al.] have provided the proof of principle that you can get these kinds of defects,” McCarrey says. “And they have added significantly to the list of chemicals that can cause these things.” However, he says the “black box” of mechanisms involved causes many to remain skeptical that such effects can occur.

To help fill knowledge gaps, Chadwick says the NIEHS will consider funding studies including those that could help define the breadth of substances that may cause such effects, determine the mechanisms involved, and evaluate factors that affect individual variation in susceptibility, such as genetic differences. Such research is especially needed since the observed effects don’t fit with current thinking that the body normally repairs epigenetic problems that occur during fetal development. This suggests unknown or modified mechanisms may be in play.

Kaylon Bruner-Tran, a Vanderbilt Univ-ersity assistant professor of obstetrics and gynecology, says these findings and others are sufficient to conclude “it is highly likely that additional biological systems will be affected by similar toxicant exposures.” Given that and the knowledge that we’ll never eliminate all the chemicals that might cause these effects, she says one research angle she’d like to see explored is how nutrition may exacerbate or mitigate any impacts: “That’s the next big issue—what do we do about the effects?”
